# ^1^H Nuclear Magnetic Resonance (NMR) Metabolomics in Rodent Plasma: A Reproducible Framework for Preclinical Biomarker Discovery

**DOI:** 10.3390/mps8040092

**Published:** 2025-08-07

**Authors:** Mohd Naeem Mohd Nawi, Ranina Radzi, Azizan Ali, Siti Zubaidah Che Lem, Azlina Zulkapli, Ezarul Faradianna Lokman, Mansor Fazliana, Sreelakshmi Sankara Narayanan, Karuthan Chinna, Mohd Fairulnizal Md Noh, Zulfitri Azuan Mat Daud, Tilakavati Karupaiah

**Affiliations:** 1 School of Biosciences, Faculty of Health and Medical Sciences, Taylor’s University, Subang Jaya 47500, Selangor, Malaysia; naeem@moh.gov.my (M.N.M.N.);; 2 Nutrition, Metabolism and Cardiovascular Research Centre, Institute for Medical Research, National Institutes of Health, Ministry of Health, Setia Alam 40170, Selangor, Malaysia; 3 Good Laboratory Practice Section, Herbal Medicine Research Centre, Institute for Medical Research, National Institutes of Health, Ministry of Health, Setia Alam 40170, Selangor, Malaysia; 4 Laboratory Animal Resource Unit, Special Resource Centre, Institute for Medical Research, National Institutes of Health, Ministry of Health, Kuala Lumpur 50588, Federal Territory of Kuala Lumpur, Malaysia; 5 Food Security and Nutrition Impact Lab, Taylor’s University, Subang Jaya 47500, Selangor, Malaysia; 6 Faculty of Business and Management, UCSI University, Kuala Lumpur 56000, Federal Territory of Kuala Lumpur, Malaysia; 7 Department of Dietetics, Faculty of Medicine and Health Sciences, Universiti Putra Malaysia, Serdang 43400, Selangor, Malaysia

**Keywords:** metabolomics, NMR, plasma, workflow, rodents

## Abstract

This protocol paper outlines a robust and reproducible framework for a ^1^H nuclear magnetic resonance (NMR) metabolomics analysis of rodent plasma, designed to facilitate preclinical biomarker discovery. The protocol details optimised steps for plasma collection in a preclinical rodent model, sample preparation, and NMR data acquisition using presaturation Carr–Purcell–Meiboom–Gill (PRESAT-CPMG) pulse sequences, ensuring high-quality spectral data and effective suppression of macromolecule signals. Comprehensive spectral processing and metabolite assignment are described, with guidance on multivariate and univariate statistical analyses to identify metabolic changes and potential biomarkers. The framework emphasises methodological rigour and reproducibility, enabling accurate quantification and interpretation of metabolites relevant to disease mechanisms or therapeutic interventions. By providing a standardised approach, this protocol supports longitudinal and translational studies, bridging findings from rodent models to clinical applications and advancing the reliability of metabolomics-based biomarker discovery in preclinical research.

## 1. Introduction

Metabolomics is rapidly becoming indispensable to translational research, particularly for animal models where biofluids such as plasma and urine enable non-invasive monitoring of systemic metabolic changes in response to genetic, dietary, or therapeutic interventions [1]. These biofluids provide real-time and time-integrated snapshots of metabolic status, making them highly valuable for longitudinal studies and bridging findings from preclinical models to clinical applications [2]. For example, plasma metabolomics in rodent models of obesity and diabetes consistently identifies disturbances in amino acid metabolism (e.g., alanine, glutamine) and energy metabolism intermediates (e.g., lactate, citrate) as hallmarks of metabolic dysfunction [3,4]. Critically, these metabolic pathways and biomarkers align closely with those observed in human non-communicable disease (NCD) cohorts, underscoring the translational relevance of rodent studies [1,5].

Amongst the analytical platforms used in metabolomics, nuclear magnetic resonance (NMR) spectroscopy is especially invaluable. NMR allows for the simultaneous detection and quantification of a broad spectrum of metabolites, whilst its non-destructive nature enables repeated analyses of the same sample, thereby offering an advantage for longitudinal sampling application [1,6,7]. The Zucker Diabetic Fatty (ZDF) rat is a well-validated preclinical model for bridging translational research in type 2 diabetes (T2D), particularly due to its recapitulation of human metabolic and pathological disease progression [8]. ZDF rats develop severe hyperglycaemia, insulin resistance, and obesity under high-fat diets, mirroring the multifactorial aetiology of human T2D [9]. Recent studies in rodent models highlight the utility of NMR-based metabolomics for capturing metabolic flexibility. For instance, non-fasted plasma sampling in Nile rats (*Arvicanthis niloticus*), a model for T2D, demonstrated stronger associations with glucose tolerance and lower replicate variance compared to fasted samples, suggesting practical advantages for metabolic phenotyping [10]. Such methodological insights enhance the reliability of plasma metabolomics in reflecting systemic metabolic states.

The integration of advanced analytical software is crucial to extracting meaningful biological insights from complex NMR datasets. Firstly, targeted quantification using tools like Chenomx NMR Suite enables a precise identification and concentration determination of metabolites in biofluids [7,11]. Furthermore, the application of multivariate statistical tools, including those implemented in SIMCA-P software (version 17), facilitate the recognition of metabolic patterns through techniques including principal component analysis (PCA) and partial least squares discriminant analysis (PLS-DA) [3]. After performing univariate analysis and robust biomarker identification, a multiple comparisons analysis is often performed to determine the groups that differ significantly [12,13,14,15]. Interpreting metabolomics data in studies involving mixed diets (e.g., whole foods or complex meals) presents challenges due to overlapping metabolic signals from multiple nutrients, interactions between dietary components, and individual variability in metabolism [16]. This contrasts with controlled interventions using single nutrients, where metabolic responses are more directly attributable to specific compounds [17]. The Benjamini–Hochberg multiple testing correction helps address these complexities by reducing false positives while maintaining sensitivity to subtle diet-related metabolic changes [18,19].

In summary, researchers can achieve reproducible and biologically relevant results in rodent models by combining standardised NMR acquisition, untargeted metabolite quantification, and integrated multivariate/univariate statistical analysis. This manuscript aims to provide a detailed, step-by-step protocol for a reproducible NMR-based metabolomics analysis of plasma in rodent models, integrating Chenomx NMR Suite (version 9) quantification, SIMCA-P (version 17) multivariate analysis, SPSS (version 26) statistical validation, and multiple testing correction. This manuscript aims to establish reproducible metabolomic workflows in preclinical research by employing a diabetic and obese rodent model, thereby bridging methodological gaps and improving the applicability of findings to human NCD studies.

## 2. Experimental Design

Twenty-four male Zucker Diabetic Fatty (ZDF *fa*/*fa*) rats (10 weeks old) and six lean male Zucker (wild-type) rats (10 weeks old) were sourced from Vital River (Beijing, China) for the experiment. ZDF rats possess a missense mutation in the leptin receptor (*Lepr*) gene, resulting in impaired leptin signalling and the spontaneous onset of obesity, insulin resistance, and T2D, as established by prior studies [20,21]. Following a 2-week quarantine, rats underwent a 1-week acclimatization under standardised conditions controlled for room temperature of 19–24 °C, 40–60% humidity, a 12/12 h light/dark cycle, *ad libitum* access to distilled water, and a standard rodent maintenance diet (11% fat, 65% carbohydrate, and 24% protein, expressed as % of total energy). Diabetes in the animal was confirmed pre-intervention via fasting blood glucose levels >200 mg/dL, as per established thresholds for diabetic phenotypes in ZDF rats [22].

ZDF rats were randomised into four groups (*n* = 6 per group), namely the control (standard rodent maintenance diet), Diet A (normal human diet), Diet B (low-carbohydrate–high-fat diet), and standard treatment (metformin, 200 mg/kg body weight daily). Lean Zucker rats (*n* = 6) were divided into two groups (*n* = 3 per group) receiving Diet A or B. After 8 weeks of intervention, rats were euthanised via 5% isoflurane inhalation, followed by cardiac puncture. Blood samples were collected into lithium heparin tubes, centrifuged (1500× *g*, 10 min, 4 °C), and plasma stored at −80 °C for NMR analysis. As the primary focus of this manuscript is on the NMR metabolomics workflow, details of the animal experimentation are therefore only briefly described.

### 2.1. Sample Size Calculation

The sample size for this study was determined using a one-way analysis of variance (ANOVA) approach, as described by [23]. The calculations utilised the degrees of freedom (DF) associated with the within-subject error term, which is defined as

$${DF}_{\;\mathrm{error}}=N-k=k(n-1)$$

where

- N: total number of subjects;
- k: number of experimental groups (*k* = 6);
- *n*: number of subjects per group.

Rearranging the formula to solve for *n* results in

$$n=\frac{{DF}_{\;error}}{k}+1$$

Steps:

1. DF Range: A DF range of 10 (minimum) to 20 (maximum) was selected to ensure statistical robustness while balancing practical constraints.
2. Per-Group Calculation:

- Minimum *n* (DF = 10):

$$n_{min}=\frac{10}{6}+1=2.7\;\approx 3\;(\mathrm{rounded}\;\mathrm{up})$$

- Maximum *n* (DF = 20):

$$n_{max}=\frac{20}{6}+1=4.3\;\approx 4\;(\mathrm{rounded}\;\mathrm{up})$$

3. Total Sample Size:

- Minimum N (*n* = 3):

$$N_{\min}=3\times 6=18\;\mathrm{rats}$$

- Maximum N (*n* = 4):

$$N_{\max}=4\times 6=24\;\mathrm{rats}$$

To ensure sufficient statistical power while accounting for potential attrition, the study employed the upper bound of the sample size calculation, allocating 6 rats per group across 4 groups of ZDF rats (6 rats × 4 groups) and 3 rats per group across 2 groups of lean Zucker rats (3 rats × 2 groups), resulting in a total sample size of 30 rats (*n* = 30).

### 2.2. Materials

1. Deuterium oxide (D_2_O) (Merck, Frankfurter Strasse 250, Darmstadt, Germany, Cat. no.: 113366).
2. Potassium dihydrogen phosphate (KH_2_PO_4_); ≥99.0% purity, (Merck, Frankfurter Strasse 250, Darmstadt, Germany, Cat. no.: 104873).
3. Sodium 3-(trimethylsilyl) propionic-2,2,3,3-d_4_ (TSP) (Sigma-Aldrich, St. Louis, MO, USA, Cat. no.: 269913).
4. Imidazole (Sigma-Aldrich, St. Louis, MO, USA, Cat. no.: I2399).
5. Sodium deuteroxide (NaOD) (Merck, Frankfurter Strasse 250, Darmstadt, Germany, Cat. no.: 372072).
6. Several 3 kDa centrifugal filters, 0.5 mL (Merck, Frankfurter Strasse 250, Darmstadt, Germany, Cat. no.: UFC500396).
7. Safe-Lock micro test tubes, 1.5 mL (Eppendorf, Hamburg, Germany, Cat. no.: 0030120086).
8. A long-form NMR pipette, tip length 9 inch (Sigma-Aldrich, St. Louis, MO, USA, Cat. no.: Z255688).
9. Several 5 mm 600 MHz NMR tubes, length 7 inch (Norell, Morganton, NC, USA, Cat. no.: 509-UP-7).
10. A 250 mL solvent bottle.
11. An NMR tube rack for 5 mm tube (Sigma-Aldrich, St. Louis, MO, USA, Cat. no.: Z118257).
12. Deionised water (for filter pre-washing).
13. Ice.
14. Dry ice.
15. Liquid nitrogen.

### 2.3. Equipment

- A mechanical pipette (Eppendorf, Hamburg, Germany).
- A long-form NMR pipette, tip length 9 inch (Sigma-Aldrich, St. Louis, MO, USA, Cat. no.: Z255688).
- A vortex mixer (IKA, Staufen, Baden-Württemberg, Germany).
- A magnetic stirrer (IKA, Staufen, Baden-Württemberg, Germany).
- A high-speed centrifuge (Kubota, Osaka, Japan).
- A −80 °C Biomedical Freezer (SANYO, Osaka, Japan).
- A liquid nitrogen tank (Chart Biomedical, GA, USA).
- A pH metre (Hanna Instruments, RI, USA).
- A 600 MHz NMR Spectrometer (JEOL, Tokyo, Japan).

### 2.4. Computational Tools

1. Chenomx NMR Suite (Chenomx Inc., Edmonton, AB, Canada).
2. SIMCA-P (Sartorius Stedim Data Analytics AB, Göttingen, Germany).
3. SPSS (IBM, Chicago, IL, USA).

## 3. Procedure

### 3.1. Sample Collection

This sample handling procedure is based on the recommendations outlined in [24], with a specific focus on minimising the processing time for serum and plasma samples.

1. Blood collection: Collect blood via a cardiac puncture into lithium heparin tubes immediately after euthanasia after the end of the experiment.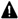 **CRITICAL STEP** EDTA or citrate tubes are not used, as these might introduce strong signals in NMR spectra, which could obscure adjacent metabolites.
2. Plasma isolation: Centrifuge blood at 1500× *g* for 10 min at 4 °C within 30 min of blood collection.
3. Storage: Aliquot plasma into 1.5 mL tubes and temporarily place on dry ice or in a liquid nitrogen tank for temporary storage. Transfer samples to −80 °C within 2–4 h of plasma separation.

### 3.2. NMR Sample Preparation

**Figure 1:**
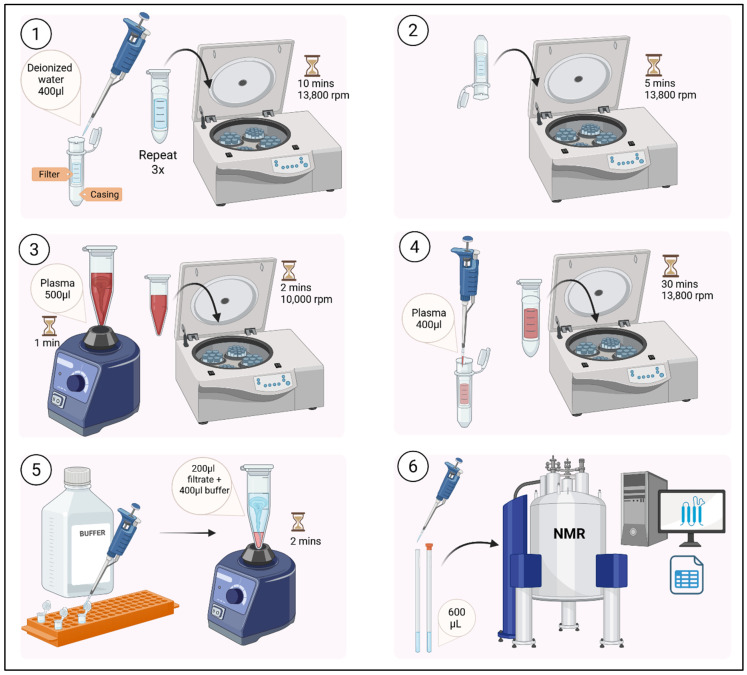
Overview of sample preparation for metabolite profiling.

This procedure is modified from the method described in [25] to suit this study’s specific requirements. A general overview of the sample preparation is shown (Figure 1).

Step 1: Thawing and preprocessing

1. Thaw frozen plasma samples on ice on the day of NMR analysis.
2. Vortex 500 μL plasma for 1 min.
3. Centrifuge at 10,000 rpm for 2 min to pellet solid debris.

Step 2: Macromolecule removal through ultrafiltration

1. Pre-wash filters to remove glycerol: Rinse 3 kDa centrifugal filters with 400 µL deionised water. Centrifuge at 13,800 rpm for 10 min for each wash. Repeat this process twice.
2. Remove residual water: Invert filters and centrifuge at 13,800 rpm for 5 min.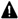 **CRITICAL STEP** Ensure there are no traces of water inside the filter and casing. Use a pipette if necessary. Filter is now ready for usage.
3. Load 400 μL plasma supernatant thawed during Step 1 onto pre-washed filters.
4. Centrifuge at 13,800 rpm for 30 min at room temperature to remove proteins/lipids.

Several studies have explored various methods for removing proteins from plasma and serum prior to NMR analysis, including solvent-based precipitation, ultrafiltration, and spectroscopic filtering [26,27,28]. Ultrafiltration is particularly advantageous for quantifying unbound, low-molecular-weight metabolites with minimal chemical artefacts. By applying a defined molecular weight cutoff, it reproducibly separates small metabolites from macromolecules like proteins and lipoproteins, improving spectral quality by reducing baseline distortion and signal overlap. Protein removal also mitigates matrix effects from differential metabolite binding, enhancing quantification consistency. Moreover, ultrafiltration avoids organic solvents, eliminating solvent-related artefacts. To address known limitations such as membrane interactions and glycerol contamination, pre-washing filters are recommended and incorporated into the current protocol [29]. While this protocol does not include a direct comparison of protein removal methods, ultrafiltration was selected based on support from the literature and practical benefits for workflow reproducibility and spectral clarity.

Step 3: Buffer preparation and dilution

1. Prepare a phosphate buffer (pH = 7.4) based on the volume required (e.g., 100 mL), with 1.232 g KH_2_PO_4_ in 100 mL D_2_O containing 100 mg TSP (0.1%) and 100 mg imidazole (0.1%). TSP functions as an internal standard, while imidazole functions as a pH indicator. The concentration of TSP in this buffer preparation is approximately 5.8 mM.
2. Use a magnetic stirrer and stirrer bar to homogenise the buffer for 5 min.
3. Add NaOD in small volumes ≈ 100 µL whilst homogenising the buffer using a magnetic stirrer for 5 min and checking the pH.
4. Repeat step 3 to achieve pH = 7.4. Wrap the solvent bottle using aluminium foil and store at 4 °C until usage.
5. Dilute filtrate with buffer in a 1:2 (*v*/*v*) ratio (e.g., 200 μL filtrate + 400 μL buffer) in a 1.5 mL tube.
6. Vortex the tube for 2 min.

TSP is a commonly used internal standard in ^1^H NMR metabolomics, employed primarily for chemical shift referencing. In this study, we use TSP due to its sharp, isolated singlet at 0.00 ppm, which provides consistent and reliable chemical shift calibration across a broad range of biofluids. Its low pKa and lack of spectral overlap with endogenous metabolites make it well-suited for direct referencing, instrument calibration, and ensuring inter-batch consistency. TSP also remains a widely adopted reference in established metabolomics protocols, supporting cross-study comparability. However, we acknowledge the well-documented limitations of TSP in protein-rich biological matrices such as serum and plasma. As reported in [30], TSP binds strongly to human serum albumin, resulting in signal attenuation, peak broadening, and chemical shift variability, particularly in untreated samples. Because only the free (unbound) fraction of TSP contributes to a sharp, quantifiable resonance, this binding reduces its reliability as an internal standard for quantification. To address this, we combine ultrafiltration [31,32,33,34] with the use of TSP at elevated concentrations (≥3 mM), as suggested in [30]. Ultrafiltration effectively removes albumin and other proteins, thereby eliminating the bound TSP fraction and yielding a sharper, more reproducible reference peak. Simultaneously, the use of a higher TSP concentration maintains sufficient free TSP levels in the filtrate for accurate referencing.

Step 4: Transfer to NMR tubes

1. Pipette 600 μL of the diluted sample into a 5 mm NMR tube using a long-form NMR pipette.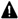 **CRITICAL STEP** Ensure that the NMR tubes are free of water which would interfere with the spectral acquisition. Use a tissue to make sure the external surface of the tube is completely dry.
2. Seal tubes and store at 4 °C until spectral acquisition using an NMR spectrometer.

### 3.3. Quality Control

To ensure the NMR spectrometer remains in optimal condition throughout the experiment, pooled quality control (QC) samples are created by mixing aliquots from all study samples. These pooled QC samples are analysed qualitatively at the start, middle, and end of each day’s run. Any instrument drift, instability, or anomalies can be promptly identified by visually inspecting the QC spectra for consistent peak positions, line shapes, and signal-to-noise ratios (SNRs). This qualitative assessment helps verify the reliability of spectral acquisition across the entire experimental sequence.

### 3.4. NMR Acquisition

The following procedure aligns with the targeted metabolomics workflow described in [35] with several modifications:

1. Instrument Setup:

- Use a 600 MHz JEOL JNM-ECZ600R NMR spectrometer (Jeol Ltd., Tokyo, Japan) maintained at 26 °C. Always use NMR tubes rated for the frequency of the spectrometer to ensure reliable, high-quality data and protect samples and the NMR instrument itself.
- Select the deuterium lock channel on the NMR spectrometer and lock onto the D_2_O signal.
- Perform shimming to optimise magnetic field homogeneity.
  - After the sample is inserted and the temperature is stabilised, the spectrometer is locked onto the deuterium resonance (^2^H) of the D_2_O solvent. The automated gradient shimming routine is applied first; this function uses the deuterium lock signal to map the magnetic field profile across the sample volume and automatically adjusts shim coil currents to correct field inhomogeneities.
  - The system uses a digital matrix shim set, which includes axial shim coils Z1 through Z6 corresponding to first- through sixth-order corrections along the *z*-axis. Specifically, Z1 compensates for linear gradients, Z2 for quadratic curvature, and Z3 to Z6 for higher-order magnetic field distortions. Following automatic shimming, the manual refinement of shim values (Z1–Z6) is carried out as needed. The adjustment sequence begins with Z1–Z3 to improve the ^2^H lock signal sharpness and continues with Z4–Z6 to correct subtle baseline distortions or peak asymmetries.
  - Throughout the process, the stability of the lock signal and the shape of a reference peak (typically the water signal at ~4.7 ppm) are monitored. Shimming is considered complete when a narrow, symmetric reference peak and stable lock level are achieved.
- Apply the presaturation-Carr–Purcell–Meiboom–Gill (PRESAT-CPMG) pulse sequence to suppress water signals and protein resonances.

2. Spectral Acquisition Parameters:

- Spectral width: 12 ppm;
- Number of scans: 64;
- Total acquisition time: 26 min;
- Time domain points: 131,072 points;
- Flip angle: 90°;
- Pre-saturation frequency: set to water resonance (≈4.7 ppm in plasma);
- Acquisition time per scan: 17.47 s;
- Relaxation delay: 7 s;
- Repetition time: 24.47 s;
- Presaturation duration: 7 s;
- CPMG tau (τ) delay: 0.19291 milliseconds;
- Number of echoes (n): 125;
- Effective echo time (2τ): 0.38582 milliseconds;
- Total echo train duration (2nτ): 48.23 milliseconds.

A repetition time of approximately 25 s is used in the acquisition protocol, which corresponds to at least five times the longest longitudinal relaxation times (T_1_) reported in the literature for the metabolites of interest [36]. This repetition time is therefore considered sufficient to allow for a near-complete recovery of longitudinal magnetization and to ensure accurate quantitative analysis. It should be noted, however, that this assessment is based on T_1_ values reported in the literature, since no inversion recovery experiments are conducted to measure T_1_ directly in the current samples. The selection of the CPMG parameters for this study, specifically a tau (τ) delay of 0.19291 milliseconds, 125 echoes, an effective echo time (2τ) of 0.38582 milliseconds, and a total echo train duration (2nτ) of 48.23 milliseconds, is guided by a review of published ^1^H NMR metabolomics studies performed in rat plasma [37,38,39]. These parameters are chosen to achieve effective suppression of broad macromolecular signals, such as those from proteins and lipoproteins, while preserving sharp signals from low-molecular-weight metabolites.

Although the general approach to CPMG implementation in rodent metabolomics is conceptually consistent, the explicit reporting of critical sequence parameters such as the tau delay and number of echoes remains inconsistent. For example, ref. [37] applies a CPMG-based spin–echo sequence in rat plasma with a fixed echo train duration of 60 milliseconds, but it does not report the tau delay or the number of echoes used. Similarly, ref. [38] reports a total echo train duration of 320 milliseconds in rat plasma using a one-dimensional spin–echo sequence, but it also omits the tau delay and number of echoes. In addition, ref. [39] applies a total echo train duration of 64 milliseconds, but again, it does not specify the tau delay and number of echoes explicitly. By contrast, CPMG protocols in human plasma are often more thoroughly reported. For instance, ref. [40] employs a tau delay of 0.83 milliseconds with 128 echoes, resulting in a total echo train duration of 212.48 milliseconds. Similarly, both refs. [41,42] use a tau delay of 0.4 milliseconds and 80 echoes, producing a total duration of 64 milliseconds. In contrast, for the current study in rodent plasma, universally agreed-upon parameters are lacking, and many published studies do not explicitly report the tau delay, echo time, or number of echoes. As such, preliminary pilot experiments are performed to optimise parameters specific to our sample system. The final values, a tau delay of 0.19291 milliseconds, 125 echoes, and a total echo train duration of 48.23 milliseconds, are empirically derived.

3. Adaptability to Different Brands

While this protocol is optimised and validated using the JEOL JNM-ECZ600R, it is adaptable to other major NMR instrument brands such as Bruker and Agilent or Varian. The fundamental CPMG parameters, such as the tau delay, number of echoes, presaturation routines, and temperature stabilisation, are universally implemented across commercial NMR platforms, enabling the core methodology to be transferred or replicated with appropriate adjustments to instrument-specific pulse sequence syntax and acquisition commands. We recommend that users carefully review their hardware specifications, including achievable tau delay increments, maximum echo counts, and probe characteristics, as these may influence effective signal suppression and data quality. Additionally, operational procedures like shimming, water suppression, and system calibration can differ between platforms. To ensure compatibility and optimal performance, we strongly suggest liaising with your respective instrument vendor when adapting this protocol to a different system. To support reproducibility and cross-platform comparability, we emphasise the importance of conducting pilot calibration runs and fine-tuning key parameters as needed. Clear documentation and complete reporting of acquisition settings are crucial, especially when comparing datasets across laboratories or instrument brands.

### 3.5. Data Preprocessing

1. Spectral Processing (Chenomx 9.0):

- Spectrum files are imported into Chenomx NMR Suite for post-acquisition processing.
- Prior to Fourier transformation, free induction decays (FIDs) are zero-filled to the next power of two if needed to improve digital resolution. The zero-filled FIDs are then Fourier-transformed to convert the time-domain signal into the frequency-domain spectrum (e.g., 131,072 points in this study), enhancing spectral resolution and enabling more precise peak identification.
- Apply an exponential line broadening (apodization) function of 0.5 Hz to enhance the SNR. Apply 1–2 Hz line broadening if the signal-to-noise ratio is critically poor and resolution loss is tolerable. Do note that broader peaks reduce integration accuracy for closely spaced resonances. Several points should be considered for selecting the degree of line broadening, as reviewed from other studies [43,44]:
  - Setting an SNR threshold for quantitation: For example, a minimum SNR of 10:1 is often recommended for reliable peak detection and integration. This threshold can be determined using reference peaks such as TSP.
  - Evaluating the risk of peak overlap: Greater line broadening is only justifiable when analyte peaks are well separated, as excessive broadening risks merging closely spaced resonances and introduces quantitation error.
  - Consistent batch processing: The same apodization parameters should be applied to all spectra within a batch, as varying these settings can artificially skew SNR measurements and introduce operator-dependent variability.
- Perform autophasing to ensure consistent and accurate phase correction across all spectra.
- Apply baseline correction using the Whittaker spline algorithm across the full spectral range, excluding the water resonance region, to effectively eliminate baseline distortions, drifts, and offsets. This approach ensures a flat and stable baseline, which is critical for accurate peak integration, reliable quantification, and robust comparison between samples.
- Reference spectra to TSP (0 ppm) for chemical shift calibration and imidazole for pH monitoring.

2. Binning and Exclusion:

- Use intelligent binning (0.04 ppm) across 0.50–12.00 ppm.
- Exclude regions:
  - Water: 4.77–4.86 ppm;
  - Imidazole: 7.29–7.33 ppm and 8.24–8.32 ppm.
- Export binned data as a non-negative integral table for multivariate analysis.

### 3.6. Multivariate Data Analysis

1. Data Preprocessing:

- Mean-centre and Pareto-scale variables (divide by √standard deviation).

2. Unsupervised Analysis (PCA):

- Perform PCA to assess clustering trends and outliers.
- Identify outliers using Hotelling’s *T*^2^ (95% confidence interval).

3. Supervised Analysis (PLS-DA):

- Conduct PLS-DA to maximise class separation.
- Validate models via CV-ANOVA (*p* < 0.05 for significance).
- Prioritise bins with VIP scores > 1.0 and visualise using S-plots.

While multivariate data analysis (MVDA) effectively identifies spectral regions of interest, it is critical to recognise that a single NMR spectral bin (e.g., 1.22 ppm) may encompass signals from multiple metabolites. Thus, MVDA alone cannot resolve structural ambiguity, necessitating targeted metabolite identification (e.g., via Chenomx spectral libraries) and quantification to confirm biological relevance and avoid misinterpretation.

### 3.7. Metabolite Identification and Quantification

The procedure below is a simplified and adapted version following the guidance outlined in the Chenomx NMR Suite V9 User Guide (Chenomx Inc., Edmonton, AB, Canada) [45].

1. Library Matching (Chenomx 9.0):

- Compare spectral peaks to the 600 MHz Human Metabolome Database (HMDB) library with pH adjustment for untargeted metabolite identification.
- Match chemical shift, multiplicity, and coupling constants based on guidelines provided by Chenomx. Fit metabolites by overlaying library spectra onto experimental data, iteratively subtracting matched peaks. Apply deconvolution to the experimental spectrum for overlapping peaks using the reference peak’s observed line shape.
- Validate fits via visual inspection of residual spectra.

2. Quantification:

- Calculate relative concentrations using peak areas normalised to TSP.
- Export results as tables for downstream analysis.

### 3.8. Statistical Analysis

1. Univariate Analysis and Multiple Testing Correction
  For studies involving group comparisons (e.g., control vs. treated), metabolites showing significant differences are first identified using a one-way ANOVA (*p* < 0.05). Post hoc pairwise comparisons are performed via Tukey’s honest significant difference (HSD) test to control family-wise error rates. The Benjamini–Hochberg procedure is applied to account for multiple hypothesis tests across metabolites, controlling the false discovery rate (FDR) at 10%. Metabolites with FDR-adjusted *p*-values < 0.05 are retained as statistically significant.
2. Workflow Integration

- ANOVA identifies metabolites with significant intergroup variation.
- Tukey-HSD pinpoints specific group differences (e.g., Diet A vs. Diet B).
- FDR correction ranks metabolites by significance and adjusts *p*-values to reduce false positives.

This hierarchical approach ensures rigorous statistical validation while maintaining interpretability in complex datasets.

### 3.9. Pathway Analysis

The procedure below is a simplified version for pathway analysis using MetaboAnalyst 6.0 [46]. This workflow is designed to guide users through the essential steps, from data preparation to result interpretation, ensuring reproducibility and clarity in metabolomics pathway analysis.

1. Define a List of Features of Interest

Identify and extract a list of metabolites that are relevant to the experimental question.

2. Perform Pathway Analysis

Use statistical methods to determine which biological pathways are significantly over-represented in your list compared to what would be expected by chance. This typically involves comparing your feature list to curated pathway databases (such as Kyoto Encyclopedia of Genomes and Genes (KEGG)) and calculating impact scores and *p*-values.

3. Visualise and Interpret Results

Visualise the impacted pathways using enriched pathways, *p*-values, impact scores, and matched metabolites to help interpret the biological significance of your findings.

## 4. Expected Results

The application of this NMR-based metabolomics protocol is expected to yield robust and reproducible metabolic profiles from rodent biofluids, enabling the systematic identification of disease-related biomarkers and pathway alterations in rodent models of obesity and diabetes. A tentative list of the detected metabolites is shown (Table 1). For each metabolite, the corresponding HMDB ID, chemical shift values (in ppm), and signal multiplicities are provided. Multiplicity notation indicates splitting patterns in the NMR spectra, with s (singlet), d (doublet), t (triplet), q (quartet), m (multiplet), and dd (doublet of doublets).

**Figure 2:**
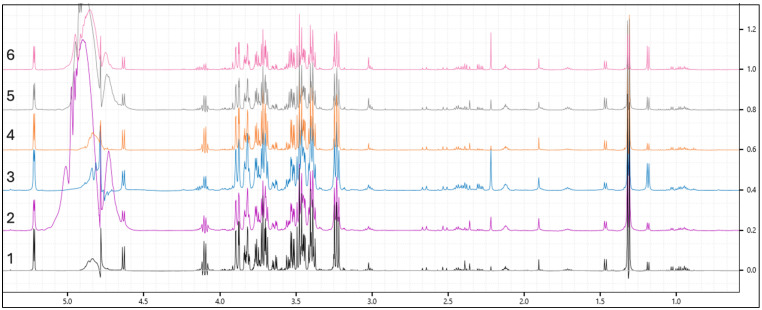
Overlay of six spectra representing each group in this study. (1—control; 2—Diet A; 3—Diet B; 4—metformin; 5—lean with Diet A; and 6—lean with Diet B).

**Figure 3:**
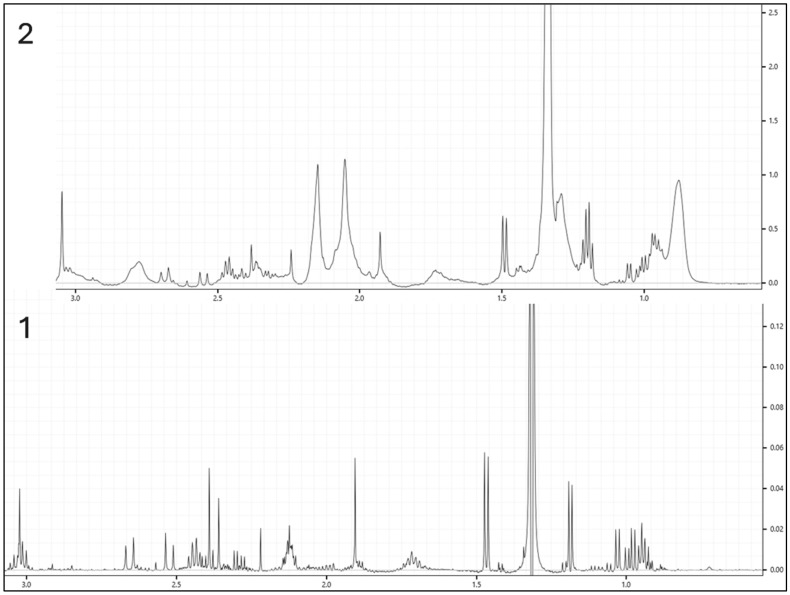
Comparison of two samples processed with and without ultrafiltration. (1—processed with ultrafiltration; 2—processed without ultrafiltration).

**Figure 4:**
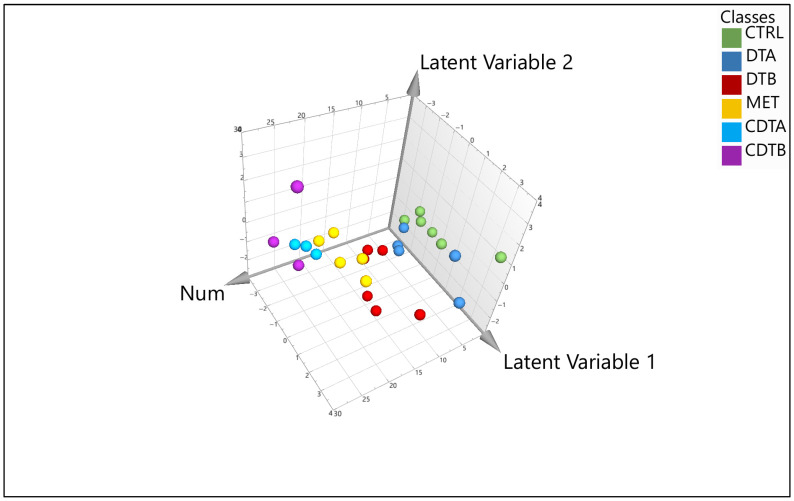
Three-dimensional (3D) PLS-DA score plot for each sample. (Green colour—control; navy blue—Diet A; red—Diet B; yellow—metformin; light blue—lean with Diet A; and purple—lean with Diet B).

Figure 2 shows the overlay of six spectra representing each group in this study. Figure 3 illustrates the differences between samples processed with ultrafiltration and without ultrafiltration. Please note that this comparison is shown for illustrative purposes only and may not represent the full variability or quantitative differences across all samples. Multivariate analysis using SIMCA-P will reveal a clear clustering of experimental groups, such as diabetic versus control animals, with PCA typically explaining more than 70% of the variance, and outliers are set to be effectively identified and excluded using Hotelling’s *T*^2^. Supervised analysis by PLS-DA is anticipated to achieve strong separation between groups (*R*^2^Y > 0.8, *Q*^2^ > 0.5), with model validity supported by significant CV-ANOVA results (*p* < 0.05). The 3D PLS-DA score plot is shown (Figure 4). Spectral bins with variable importance in projection (VIP) scores greater than 1.0 will emerge as key discriminators between phenotypes.

Metabolite identification and quantification using the Chenomx NMR Suite will enable a confident annotation of key metabolites, including branched-chain amino acids (leucine, valine), lactate, succinate, citrate, and glucose, with characteristic chemical shifts and spectral regions. The protocol’s preprocessing steps will ensure the exclusion of confounding signals, such as residual water and imidazole, resulting in clean and interpretable spectra. Statistical validation will reveal that numerous metabolites exhibit significant intergroup differences by an ANOVA, with post hoc Tukey-HSD tests pinpointing specific pairwise group differences. Following Benjamini–Hochberg correction for multiple tests (FDR = 10%), a subset of metabolites will retain statistical significance, reducing the risk of false positives. Biologically, the protocol is expected to capture hallmark metabolic changes associated with NCDs. Quality control samples will demonstrate high reproducibility, with coefficients of variation below 5% for major metabolites, and the use of intelligent binning will ensure consistent integration and minimal technical variability across replicates. Overall, these results will substantiate the protocol’s effectiveness for comprehensive, statistically rigorous metabolomic profiling in preclinical rodent studies. This protocol establishes a reproducible NMR-based metabolomics workflow for rodent biofluids, enabling a robust identification of metabolic alterations relevant to obesity and diabetes. The protocol bridges preclinical and clinical research by integrating standardised NMR acquisition, multivariate analysis, and rigorous statistical validation, which supports the discovery of key metabolic biomarkers and therapeutic targets.

## 5. Conclusions

These findings demonstrate the robustness and applicability of the described NMR-based metabolomics workflow for translational research, especially in preclinical rodent models of metabolic disease. The standardised methodology enables reproducible detection and quantification of clinically relevant metabolites, facilitating mechanistic insights and biomarker discovery. While optimised on the JEOL JNM-ECZ600R spectrometer, this workflow is adaptable to other major platforms such as Bruker, Agilent, and Varian through appropriate adjustments of instrument-specific parameters. Users should carefully consider hardware capabilities and operational differences, perform pilot calibrations, and thoroughly document acquisition settings to ensure reproducibility and cross-platform comparability. Looking ahead, adapting this workflow to additional sample matrices such as faecal material or incorporating stable isotope tracing techniques could provide a deeper understanding of complex biological processes, including gut–liver axis interactions and alterations in metabolic flux. It is important to note that this protocol does not include a comparative evaluation of different protein removal techniques or the utilisation of alternative experiments to assess their effects relative to the CPMG sequence. Such comparisons could offer valuable insights into optimising sample preparation and spectral acquisition tailored to specific research questions. Nevertheless, it is important to acknowledge the inherent limitations of NMR spectroscopy. Compared with mass spectrometry, NMR exhibits lower sensitivity and may encounter challenges due to spectral overlap in complex biological mixtures, limiting the detection of low-abundance and protein-bound metabolites. Therefore, the integration of NMR with complementary analytical platforms offers substantial potential for overcoming these constraints and achieving a more comprehensive, multidimensional metabolic profile. By combining the quantitative reliability and structural elucidation capabilities of NMR with the sensitivity and coverage of other techniques, future studies can leverage a synergistic approach that enhances metabolomic insights relevant to both preclinical and clinical settings.

## Figures and Tables

**Table 1:** Identified metabolites.

Metabolite	HMDB ID	Chemical Shift (Multiplicities)
1,6-Anhydro-β-D-glucose	HMDB00640	3.5 (m), 3.7 (m), 4.1 (m), 4.6 (d), 5.4 (d)
2-Hydroxyvalerate	HMDB01863	0.9 (t), 1.3 (d), 1.4 (m), 1.6 (m), 1.7 (m), 4.0 (dd)
2-Methylglutarate	HMDB00422	1.1 (d), 1.6 (m), 1.7 (m), 2.1 (m), 2.2 (m)
2-Oxoglutarate	HMDB00208	2.4 (s), 3.0 (s)
3-Aminoisobutyrate	HMDB03911	1.2 (dd), 2.6 (dd), 3.0 (s), 3.1 (s)
3-Hydroxybutyrate	HMDB00357	1.2 (d), 2.3 (dd), 2.4 (m), 4.1 (d)
Acetate	HMDB00042	1.9 (s)
Agmatine	HMDB01432	1.7 (m), 3.0 (m), 3.2 (s), 7.2 (s)
Alanine	HMDB00161	1.5 (d), 3.8 (q)
Anserine	HMDB00194	2.6 (m), 2.7 (m), 3.0 (s), 3.2 (s), 3.8 (m), 4.5 (d), 7.1 (s), 8.2 (s), 8.3 (s)
Cadaverine	HMDB02322	1.5 (t), 1.7 (m), 3.0 (t)
Carnitine	HMDB00062	2.4 (t), 3.2 (s), 3.4 (s), 4.6 (d)
Choline	HMDB00097	3.2 (s), 3.5 (t), 4.1 (m)
Citrate	HMDB00094	2.5 (s), 2.7 (s)
Creatine	HMDB00064	3.0 (s), 3.9 (s)
Formate	HMDB00142	4.4 (s)
Gluconate	HMDB00625	3.7 (m), 3.8 (m), 4.0 (m), 4.1 (m)
Glucose	HMDB00122	3.2 (m), 3.4 (m), 3.5 (m), 3.7 (m), 3.8 (m), 3.9 (m), 4.6 (d), 5.2 (d)
Glutamate	HMDB00148	2.0 (m), 2.1 (m), 2.3 (t), 2.4 (t), 3.7 (dd)
Glutamine	HMDB00641	2.1 (t), 2.4 (m), 2.5 (dd), 3.8 (q), 6.9 (d), 7.6 (d)
Glycine	HMDB00123	3.6 (s)
Glyclyproline	HMDB00721	1.8 (m), 1.9 (m), 2.0 (m), 2.1 (m), 2.2 (m), 2.3 (m), 3.5 (m), 3.6 (m), 3.9 (m), 4.3 (m)
Histidine	HMDB00177	3.1 (d), 3.2 (d), 4.0 (t), 7.1 (d), 7.9 (d)
Homocysteine	HMDB00742	2.1 (m), 2.2 (m), 2.6 (dd), 2.7 (m), 3.9 (s)
Hydroxyacetone	HMDB06961	2.1 (s), 4.4 (s)
Isoleucine	HMDB00172	0.9 (d), 1.0 (d), 1.2 (t), 1.5 (m), 2.0 (m), 3.7 (m)
Lactate	HMDB00190	1.3 (d), 4.1 (q)
Leucine	HMDB00687	0.9 (d), 1.0 (d), 1.7 (m), 3.7 (m)
Lysine	HMDB00182	1.4 (m), 1.5 (m), 1.7 (m), 1.9 (m), 3.0 (t), 3.7 (m)
Methionine	HMDB00696	2.1 (m), 2.2 (m), 2.6 (t), 3.9 (s)
N-Methylhydantoin	HMDB03646	2.9 (s), 4.1 (s)
Ornithine	HMDB00214	1.7 (dd), 1.8 (m), 1.9 (t), 3.1 (q), 3.8 (dd)
Proline	HMDB00162	2.0 (m), 2.1 (m), 2.3 (m), 3.3 (m), 3.4 (m), 4.1 (m)
Putrescine	HMDB01414	1.8 (t), 3.0 (t)
Pyruvate	HMDB00243	2.4 (s)
Succinate	HMDB00254	2.4 (s)
Taurine	HMDB00251	3.3 (t), 3.4 (t)
Threonine	HMDB00167	1.3 (d), 3.6 (m), 4.3 (d)
Trimethylamine N-oxide	HMDB00925	3.3 (s)
Tyrosine	HMDB00158	3.0 (s), 3.2 (s), 3.9 (m), 6.9 (d), 7.2 (d)
Valine	HMDB00883	1.0 (d), 2.3 (m), 3.6 (m)
myo-Inositol	HMDB00211	3.3 (t), 3.5 (t), 3.6 (t), 4.1 (m)
trans-4-Hydroxy-L-proline	HMDB00725	2.1 (m), 2.4 (m), 3.4 (m), 3.5 (m), 4.3 (d), 4.7 (d)
π-Methylhistidine	HMDB00479	3.2 (s), 3.3 (s), 3.7 (m), 4.0 (t), 7.1 (d), 8.0 (d)

Notes: (s)—singlet; (d)—doublet; (t)—triplet; (q)—quarter; (m)—multiplet; and (dd)—doublet of doublets.

## Data Availability

The raw data supporting the conclusions of this article will be made available by the authors upon reasonable request. A proposal with a detailed description of study objectives and a statistical analysis plan will be needed for an assessment of requests. Additional materials might also be required during the process of assessment.

## Abbreviations

The following abbreviations are used in this manuscript:

3D	Three-dimensional
ANOVA	Analysis of variance
CPMG	Carr–Purcell–Meiboom–Gill
DF	Degrees of freedom
FDR	False discovery rate
FID	Free induction decay
HMDB	Human Metabolome Database
HSD	Honest significant difference
KEGG	Kyoto Encyclopedia of Genomes and Genes
MVDA	Multivariate data analysis
NCD	Non-communicable disease
NMR	Nuclear magnetic resonance
PCA	Principal component analysis
PLS-DA	Partial least squares discriminant analysis
PRESAT	Presaturation
QC	Quality control
SNR	Signal-to-noise ratio
T2D	Type 2 diabetes
TSP	Sodium 3-(trimethylsilyl) propionic-2,2,3,3-d_4_
VIP	Variable importance projection
ZDF	Zucker diabetic fatty
